# Hibernation-like state induced by an opioid peptide protects against experimental stroke

**DOI:** 10.1186/1741-7007-7-31

**Published:** 2009-06-17

**Authors:** Cesar V Borlongan, Teruo Hayashi, Peter R Oeltgen, Tsung-Ping Su, Yun Wang

**Affiliations:** 1 National Institutes of Health, National Institute on Drug Abuse Intramural Research Program, Cellular Neurobiology Branch, Baltimore, MD, USA; 2 Department of Pathology, University of Kentucky, Lexington, KY, USA

## Abstract

**Background:**

Delta opioid peptide [D-ala2,D-leU5]enkephalin (DADLE) induces hibernation in summer ground squirrels, and enhances preservation and survival of isolated or transplanted lungs and hearts. In the present study, we investigated the protective effect of DADLE in the central nervous system.

**Results:**

Adult Sprague-Dawley rats were pretreated with DADLE (4 mg/kg every 2 h × 4 injections, i.p.) or saline prior to unilateral occlusion of the middle cerebral artery (MCA). Daily behavioral tests revealed that ischemic animals treated with DADLE did not show any significant behavioral dysfunctions compared with saline-treated ischemic animals. Opioid antagonists only transiently inhibited the protective effect of DADLE, indicating the participation of non-opioid mechanisms in DADLE neuroprotection. Histological examination using triphenyltetrazolium chloride (TTC) revealed that brains from ischemic animals treated with DADLE, either alone or with adjuvant opioid blockers, exhibited almost completely intact striata. In contrast, brains from ischemic animals that received saline showed significant infarction in the lateral striatum. Analyses of apoptotic cell death revealed a significant increase in the p-53 mRNA expression in the striatum of ischemic animals that received saline, while those that received DADLE exhibited near normal striatal p-53 expression. This protective effect was accompanied by significant increments in protein levels of glial cell line-derived neurotrophic factor in the striatum of DADLE-treated ischemic animals.

**Conclusion:**

These results indicate that DADLE protected against necrotic and apoptotic cell death processes associated with ischemia-reperfusion injury. The present study demonstrates that delta opioids are crucially involved in stroke, suggesting that the opioid system is important in the study of brain injury and protection.

## Background

Opioids have been well-documented as drugs of abuse [1] and as potential analgesics [2]. Opioid receptors in tissues and organs may mediate the drug activity, thus opioids acting preferentially on mu, kappa or delta receptor alone may produce unique behavioral and physiological responses. Delta opioids have been recently implicated in ischemia [3-5]. Following occlusion of the middle cerebral artery in mice, delta binding sites were decreased at least 6 h earlier than reductions in mu or kappa binding sites, and concomitant with the extension of the infarct core [3]. The early reduction in delta receptors prior to any observable brain damage suggests that these receptors are the class most sensitive to brain insults. Accordingly, stimulating the delta receptors may promote anti-ischemic effects. A 'natural hibernation' condition has been suggested to achieve such anti-ischemic effects [4,5]. The preservation of isolated rat hearts can be improved by pharmacologically duplicating the common pathway to natural hibernation and ischemic preconditioning, that is, through an opening of the ATP-sensitive potassium channels [4]. Brain tissues collected from hibernating ground squirrels were more tolerant to hypoxia and aglycemia than those tissues from active squirrels [5]. These results indicate that drugs that induce hibernation, such as [D-ala2,D-leU5]enkephalin (DADLE), could act as anti-ischemic agents.

The commonality in the mechanisms (for example, oxidative stress, free radical formation) underlying the survival/degeneration of tissues in the periphery and in the central nervous system (CNS) suggests that DADLE may protect against cerebral ischemia. In the present study, we examined this protective effect of DADLE in the CNS by pretreating young adult rats with this peptide and subsequently exposing them to occlusion and reperfusion surgery of the middle cerebral artery (MCAor). Routine behavioral tests were employed to characterize functional alterations during an acute post-ischemia period. The volume and size of infarction were analyzed using triphenyltetrazolium chloride (TTC) [6], a histological assay for determining dehydrogenase activity. Because apoptotic mechanism of cell death accompanies the MCA or stroke model [7,8], we also investigated whether DADLE could alter the expression of p-53 mRNA, which is an associated marker for apoptosis. Finally, to elucidate possible opioid receptor mediation of DADLE's functions in the CNS, we carried out pharmacologic manipulations using delta opioid antagonists naltrexone (a universal opioid receptor blocker) and naloxone methaiodide (a peripheral opioid receptor blocker).

## Results

### DADLE attenuates motor asymmetrical behaviors

**Figure 1:**
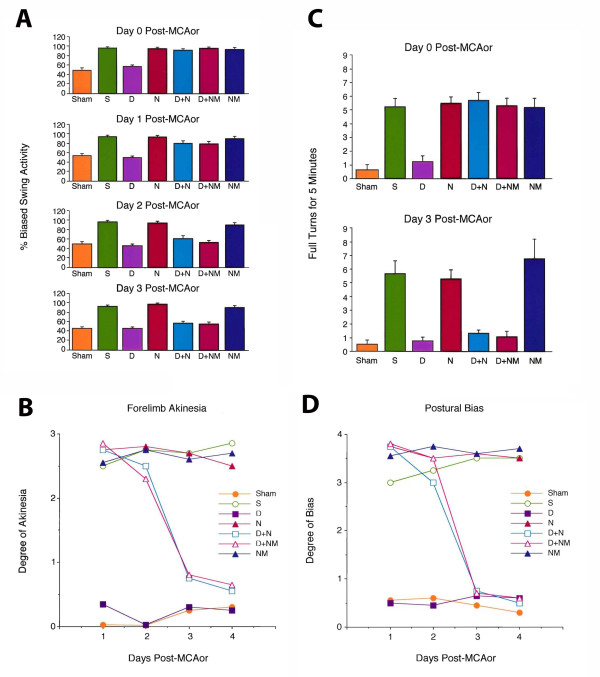
**[D-ala2,D-leU5]enkephalin (DADLE) attenuates ischemia-induced asymmetrical motor deficits**. Ischemic animals pretreated with saline (S), naltrexone alone (N) or naloxone methiodide alone (NM) displayed significant biased swing activity (panel A), postural bias (panel B), spontaneous rotational behavior (panel C), and forelimb akinesia (panel D). In contrast, ischemic animals pretreated with DADLE + naltrexone (D + N) or DADLE + naloxone methiodide (D + NM) exhibited significant dysfunctions in the same behavioral tests only at 0 to 24 h post occlusion and reperfusion surgery of the middle cerebral artery (MCAor) (days 0 and 1), and thereafter showed near normal behaviors at 48 h and 72 h post MCAor (days 2 and 3). In contrast, ischemic animals pretreated with DADLE alone (D) displayed near normal behaviors throughout the post-MCAor test period.

Daily behavioral tests revealed that ischemic animals treated with DADLE displayed near normal behaviors throughout the post-MCAor test period in a battery of tests (Figure 1). In contrast, ischemic animals pretreated with saline, naltrexone alone or naloxone methiodide alone displayed significant abnormalities in elevated body swing test (EBST), spontaneous rotational test, postural bias test, and forelimb akinesia test throughout the post-MCAor test period. The mean locomotor deficits in these ischemic animals are as follows: 82.8% ± 12.3 (mean percentage biased swing response), 2.52 ± 0.56 (mean ipsiversive rotations per minute), 2.25 ± 0.31 (mean postural bias score), and 1.17 ± 0.15 (mean akinesia score). Ischemic animals pretreated with DADLE + naloxone methiodide or DADLE + naltrexone also exhibited similar behavioral deficits at 24 h post-MCAor, but showed near normal behaviors at 48 h and 72 h post-MCAor. This observation of transient behavioral abnormalities in ischemic animals treated with DADLE + opioid antagonists suggests that DADLE only partially exerted its protection via the opioid system, and non-opioid mechanisms appear to mediate the majority of protective effects of DADLE.

### DADLE reduces cerebral infarction

**Figure 2:**
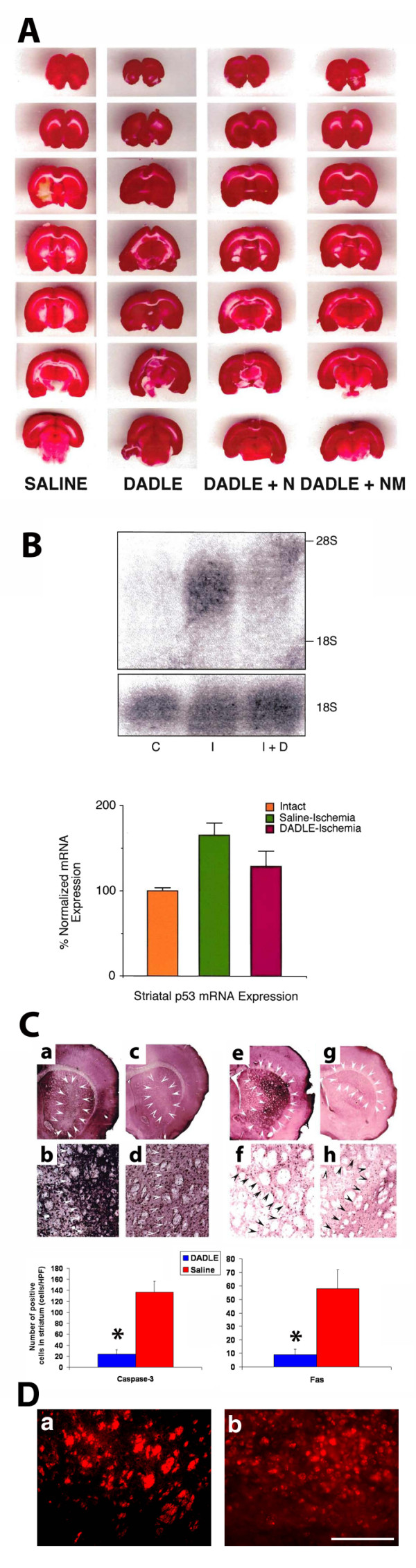
**[D-ala2,D-leU5]enkephalin (DADLE) reduces necrotic and apoptotic (panel B) cell death associated with ischemia**. **(A) **Triphenyltetrazolium chloride (TTC) staining at 72 h post occlusion and reperfusion surgery of the middle cerebral artery (MCAor) revealed that the striata from ischemic animals exposed to ischemia/reperfusion surgery and pretreated with saline showed dehydrogenase-deficient tissue (negative TTC stains). In contrast, the striata from ischemic animals pretreated with DADLE, DADLE + naloxone methiodide, or DADLE + naltrexone did not reveal any dehydrogenase deficiency. **(B) **Ischemic animals exposed to ischemia/reperfusion injury and treated with saline showed a significant increase in mRNA expression of p-53 in their ischemic striata (65% increment compared with intact striatum of normal, control animals) at 24 h after stroke surgery. In contrast, ischemic animals treated with DADLE exhibited only a small increment in p-53 mRNA expression in their ischemic striata at the same time period (28% increment compared with intact striatum of normal, control animals; not significantly different from control values). Comparisons of ischemic striata between these two groups showed a marked reduction in p-53 mRNA expression in DADLE-treated ischemic animals compared with saline-treated ischemic animals. **(C) **Immunohistochemical analyses of phenotypic markers of apoptosis revealed that DADLE significantly reduced caspase-3- (panels a, b, c, d) and Fas-positive cells (panels e, f, g, h) in DADLE-treated ischemic animals compared to saline-treated ischemic animals. Quantitative data shown in bar graphs and represent means ± S.E.M. Asterisks correspond to statistical significance at *P <*0.05. To better capture the necrotic and apoptotic cells in saline-treated ischemic animals (panel d), higher magnification images are generated from propidium iodide (a) and caspase-3 (b) immunofluorescently labeled striatal cells, respectively. Scale bar = 50 μm (a), 100 μm (b).

TTC staining at 24 or 72 h after reperfusion revealed that brains from ischemic animals that were treated with DADLE, alone or with adjuvant opioid blockers, had almost completely intact striata, whereas those from ischemic animals that received saline showed significant infarction in the lateral striatum (Figure 2A). Ischemic animals pretreated with saline had a mean volume (± S.E.M.) of 81.2 ± 5.3 mm^3 ^of infarcted striatal tissue, while ischemic animals pretreated with DADLE alone, DADLE + naltrexone, or DADLE + naloxone methiodide had no detectable infarction. Ischemic animals that received naltrexone alone or naloxone methiodide alone had a mean volume of 78.7 ± 7.4 mm^3 ^of striatal infarcted tissue, which did not differ from the ischemic animals that received saline alone. Analysis of variance (ANOVA) revealed significant treatment effects (*P *< 0.0001) and *post hoc *t-tests revealed significant reduction in infarct volume in DADLE-treated ischemic animals, including those co-administered with opioid blockers, compared with saline-treated ischemic animals (*P *< 0.05). These histological observations were consistent for both time periods of histological examination. The observation of almost intact striatum following MCAor in DADLE-treated animals, even with the co-administration of opioid antagonists, suggests that DADLE protected against necrotic cell death processes induced by ischemia-reperfusion injury.

### DADLE decreases ischemia-induced apoptotic cell death

Analyses of apoptotic cell death revealed a significant increment in the mRNA expression of p-53 in the striatum of ischemic animals that received saline, while those that received DADLE exhibited near normal striatal p-53 expression. ANOVA revealed a significant difference across treatment conditions (*F*2,15 = 5.6, *P *< 0.05) (Figure 2B). Normalized values of p-53 mRNA expression showed that vehicle-treated ischemic animals had a significant increase in mRNA expression of p-53 in the ischemic striatum (65% increment) at 24 h post-MCAor surgery compared with the intact striatum of control, normal animals (*P *< 0.01). In contrast, there was no significant difference in p-53 mRNA expression between the ischemic striatum (28% increment) of DADLE-treated ischemic animals and the intact striatum of control, normal animals (*P > *0.05). Comparisons of ischemic striata showed a marked reduction (but only a trend, *P *= 0.07) in p-53 mRNA expression in DADLE-treated ischemic animals compared with vehicle-treated ischemic animals. Moreover, immunohistochemical analyses of phenotypic markers of apoptosis revealed significant reductions in caspase-3- and Fas-positive cells in DADLE-treated ischemic animals compared with vehicle-treated ischemic animals (Figure 2C). These results indicate that DADLE protected against apoptotic cell death processes associated with ischemia-reperfusion injury.

### DADLE increases levels of glial cell line-derived neurotrophic factor expression

**Figure 3:**
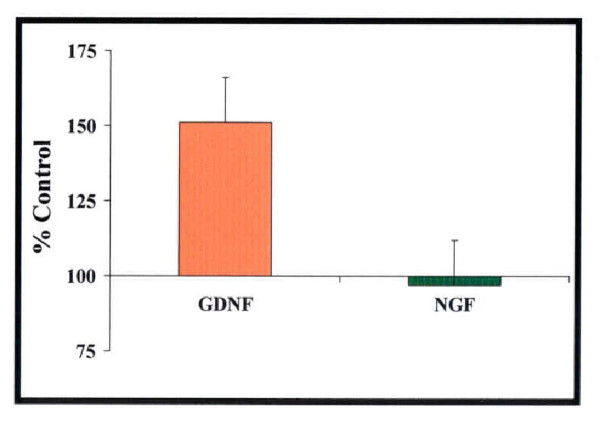
**[D-ala2,D-leU5]enkephalin (DADLE) increases expression of glial cell line-derived neurotrophic factor (GDNF) in brain tissues**. Enzyme-linked immunosorbent assays (ELISA) revealed that the levels of GDNF proteins, but not nerve growth factor (NGF), were significantly higher in striatal and cortical tissues harvested from ischemic animals treated with DADLE compared with those treated with saline. Data are expressed as mean percent of control ± S.E.M. Asterisk indicates *P <*0.05. *n *= 6 samples for each neurotrophic factor examined.

Enzyme-linked immunosorbent assay (ELISA) examination of expression of neurotrophic factors revealed elevated levels of glial cell line-derived neurotrophic factor (GDNF), but not nerve growth factor (NGF), in the striatal and cortical tissues harvested from ischemic animals treated with DADLE (Figure 3). Ischemic animals treated with DADLE had significantly higher levels of GDNF compared with those treated with saline (*P <*0.05). However, both groups did not differ in their levels of NGF (*P > *0.05). These results indicate that DADLE specifically increased GDNF, but not NGF.

## Discussion

The present study demonstrated that DADLE protected against MCA or ischemia-induced deficits. Animals that were pretreated with DADLE and subsequently exposed to the MCAor surgery did not show significant behavioral deficits as revealed by a battery of tests, while ischemic animals pretreated with saline alone exhibited significant dysfunctions in all behavioral tests. Delta opioid blockers naltrexone and naloxone methaiodide transiently antagonized the protective effects of DADLE at 24 h post MCAor but not thereafter, indicating that DADLE's effects are only partially mediated by opioid receptors. The data on TTC staining (a marker of irreversible cell damage) revealed almost no detectable dehydrogenase-deficient tissue (necrotic infarction) in the striatum (the ischemic core) at 24 h or 72 h post MCAor in ischemic animals treated with DADLE alone or DADLE + opioid blockers. Whereas opioid blockers transiently suppressed the behavioral protection by DADLE, they did not antagonize DADLE's inhibition of ischemia-induced necrosis. In addition, striatal p-53 mRNA expression, an index of apoptosis, was significantly suppressed by DADLE at 24 h post MCAor. In contrast, an increment in striatal p-53 mRNA expression was noted in ischemic animals that received saline alone. Thus, pretreatment with DADLE was shown to rescue both ischemia-induced necrotic and apoptotic cell injury, which could have promoted the observed behavioral protection.

The observation that DADLE exerts therapeutic effects in a stroke model provides evidence for the need for further investigating the role of the opioid system in cell degeneration and regeneration. The novelty of the present study is the demonstration that a delta opioid agonist drug that can induce hibernation protected the brain from ischemic damage. The mu opioid, morphine, has been recently shown to induce a Fas-mediated cell death [9]. Our data, however, indicate that delta opioids possess protective effects against cell death, for both necrotic and apoptotic types. These results suggest that opioids, depending on the specific receptor they trigger, can promote either degenerative or protective effects, in that mu opioids may promote neurotoxic effects [9], while kappa [10,11] and delta opioids may produce protective/restorative effects.

The significant reductions in cerebral infarction and apoptotic cell death markers (p-53 mRNA expression, caspase-3, and Fas) indicate that DADLE centrally promoted its protective effects. We recently demonstrated that DADLE blocks and even reverses the loss of dopamine transporters induced by chronic methamphetamine treatment [12,13]. Moreover, DADLE inhibits accumulations of superoxide anions and hydroxyl radicals [14], which have been shown as exacerbating factors for many neurological disorders, including stroke [15,16]. The possibility exists that ischemia-induced cell death involves the dopaminergic system, specifically the striatal dopamine pathway. Indeed, we reported that many functional deficits associated with the MCAor are striatal dopamine-mediated behaviors [17]. Animals with striatal ischemia display methamphetamine-induced rotational behaviors [18], as well as impairments in passive avoidance and Morris water maze tests [19]. These findings implicate the dopamine-rich innervated striatum as a critical brain area for initiating neuroprotective strategies in this stroke model.

The mechanism we proposed for the observed protective effects of DADLE is the ability of DADLE to increase expression of GDNF in the striatum and cortex, which are the MCAor stroke target brain areas. We previously demonstrated that intracerebral infusion of GDNF protects against cerebral ischemia [6]. Moreover, it has been established that GDNF is a highly selective dopamine neuron survival agent [20,21]. We recently reported that DADLE enhances embryonic dopamine cell viability *in vitro *and following intrastriatal transplantation [22] and protects adult dopaminergic neurons *in vivo *against 6-hydroxydopamine-induced neurotoxicity [23]. The present observation of increased GDNF striatal levels following DADLE treatment suggests that the striatal dopaminergic system is a highly potent target for DADLE in reversing ischemia cell injury, as well as other diseases characterized by dopamine dysfunctions.

The different onsets of behavioral recovery between ischemic animals that received DADLE and those treated with DADLE and opioid receptor blockers deserve clarification. DADLE-treated ischemic animals displayed significantly less behavioral deficits in the battery of tests throughout the post-MCAor test period. In contrast, those ischemic animals that received a combination of DADLE and opioid receptor blockers did not exhibit improvements in stroke-induced behavioral deficits until 48 h, which persisted up to 72 h post MCAor. This observation of transient behavioral abnormalities in ischemic animals treated with DADLE and opioid antagonists suggests that DADLE only partially exerted its protection via the opioid system, and non-opioid mechanisms appear to mediate the majority of protective effects of DADLE. Accordingly, blocking DADLE via the opioid pathway should only partially prevent DADLE's therapeutic benefits. The partial antagonistic effects of opioid blockers were reflected in the delayed onset of behavioral recovery in ischemic animals treated with DADLE and opioid blockers. The question arises then of why recovery could be initiated at a later post-MCAor time point if these DADLE + opioid blocker treated ischemic animals already exhibited behavioral deficits at early post-MCAor phase. Such delayed functional recovery is likely explained by the temporal profile of DADLE's non-opioid therapeutic action (that is, GDNF upregulation). Based on the reported neurotrophic effects of GDNF on endogenous cells [24,25], it is possible that recruitment of newly formed cells from neurogenic sites (that is, subventricular zone) towards the ischemic striatal area was delayed, thereby corresponding to the delayed behavioral recovery. Recent data from our laboratory indicate DADLE robust induction of neurogenesis (data not shown).

The present behavioral and histological results parallel the reported evolution of the penumbra during the acute phase of ischemic stroke [26,27]. Stroke-induced histological alterations as revealed by TTC staining reveals that DADLE-treated ischemic animals, as well as those that received combined DADLE and opioid blockers, all demonstrate near absent infarcts at 24 h post-stroke (that is, the earliest time point we performed such infarct evaluation). Despite the absence of massive infarcts, ischemic animals treated with DADLE and opioid blockers initially exhibited significant behavioral deficits at 24 h, then their behaviors eventually improved at 48 h and remained near normal levels at 72 h post-stroke. Notwithstanding near normal gross histology, the behavioral deficits at the very acute stage of stroke could be due to subtle lesions and/or apoptotic cell death not detected by TTC staining. The evolution of ischemic penumbra in these animals that initially demonstrated behavioral deficits could have been similarly abrogated by DADLE's non-opioid therapeutic action. It is likely that DADLE-mediated GDNF upregulation had already been in place even prior to stroke, since DADLE was administered repeatedly starting at 6 h pre-stroke. Our pre-stroke DADLE paradigm is in agreement with previous studies reporting that optimal GDNF therapeutic effects are achieved when initiated at an ample time interval prior to injury [28,29]. DADLE neutrophic effects, even in those animals co-treated with opioid blockers, could have been manifested in a two-pronged approach. One pathway is to combat directly the ischemic penumbra, and another process discussed above is via recruitment of endogenous stem cells. The combination of these two neuroprotective mechanisms was robust in DADLE treated ischemic animals, while delayed (that is, 48 h) for those that received combined DADLE and opioid blockers. Clearly, additional studies are warranted to reveal the exact mechanism of action of DADLE, but our results concur with prevailing concepts on stroke cell death cascades and neuroprotective pathways.

## Conclusion

The present study demonstrated that DADLE, possibly by increasing GDNF expression in critical brain regions, prevented cell death processes and behavioral abnormalities associated with experimental stroke in rats. The observation that a delta opioid peptide, previously shown to induce hibernation, attenuated deficits inherent in cerebral ischemia provides a new pharmacological target for stroke therapy.

## Methods

### Subjects

**Figure 4:**
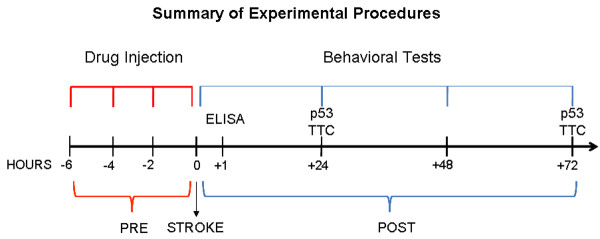
**Schematic diagram summarizing timeline of experimental procedures in this study**.

A total of 56 male Sprague-Dawley rats (Charles River, IN, USA), weighing between 290 g and 330 g, served as subjects in the present study. The animals were housed in a temperature-controlled room with normal 12–12 h light-dark cycle. Food and water were freely available in the house cage. All animal handling and surgical procedures adhered to NIH IACUC guidelines. All investigators directly involved in drug treatments, behavioral testing, and immunohistochemical analyses were blinded to the treatment conditions. A schematic diagram is provided summarizing experimental procedures in this study (Figure 4).

### Treatment conditions

To establish the protective property of DADLE in the central nervous system, we pretreated young adult Sprague-Dawley rats with DADLE (4 mg/kg every 2 h × 4 injections, i.p.) or saline prior to unilateral MCAor. To elucidate the opioid mediation on the effects of DADLE, some ischemic animals were treated with naltrexone (a universal opioid receptor blocker) or naloxone methiodide (a peripheral opioid receptor blocker). Animals were randomly assigned to one treatment condition: DADLE alone (*n *= 10), DADLE + naltrexone (*n *= 8), DADLE + naloxone methiodide (*n *= 8), naloxone methiodide alone (*n *= 8), naltrexone alone (*n *= 8), saline vehicle alone (*n *= 8), and normal, no surgery/no treatment (*n *= 6). The present dosage of DADLE was within the reported efficacious range for protection against loss of dopamine transporter induced by chronic methamphetamine [13,14]. The dosages for naltrexone (0.1 mg/kg, i.p.) and naloxone methiodide (0.01 mg/kg, i.p.) were based on previous studies showing each drug's maximal antagonistic effects [30,31]. Each delta opioid antagonist was injected simultaneously with DADLE.

### Ischemia surgery

Following the last drug injection, animals were subjected to MCAor surgery. The surgical procedures were done under aseptic conditions. Throughout the surgery and until recovery, body temperature was kept at 37°C using a thermal blanket connected to a rectal probe that controlled the heat delivery to the animal. In the present study, we used the MCAor embolic technique as described elsewhere [17]. Under deep anesthesia using chloral hydrate (400 mg/kg, i.p.), the right common carotid artery was identified and isolated through a ventral midline cervical incision. We used a suture filament with its tip coated with a combination of dental substance mixture that allows not only a smooth finish (avoiding artery perforations during insertion into the lumen) but more importantly a customized tip tapered to the desired gauge depending on the animal weight, age, and gender. Such a highly customized filament tip blocks the MCA better than a flamed tip, allowing us to achieve better and consistent stroke and thus development of infarct within 1 h faster than conventional flame-tapered filaments requiring 90 or 120 min of occlusion. Furthermore, TTC and laser Doppler measurements revealed that our 1-h MCAo produces infarct size and regional cerebral blood flow reduction (>80%) comparable to that achieved by 90- or 120-min MCA occlusion. About 15 to 17 mm of the suture filament was inserted from the junction of the external and internal carotid arteries to block the MCA. The right MCA was occluded for 1 h. Based on our own studies and several others, a 1-h occlusion of the MCA results in maximal infarction [17]. A major factor we highlight here that could have led to the present infarcts limited to the striatum is the use of liquid anesthesia, as opposed to gas anesthesia. Since all animals received the same anesthesia, any differences in stroke outcome parameters across could be ascribed to the experimental drug.

### Behavioral tests

After recovery from anesthesia, animals were evaluated on a battery of tests including spontaneous rotational test, EBST, postural bias test, and forelimb akinesia test. We have reported that animals with successful MCA occlusion exhibit asymmetric behaviors [17]. Ischemic animals have been observed to rotate >2 full 360° ipsiversive turns, swing = 75% towards the ischemic side, exhibit a postural deficit characterized by a clipped (to the chest) left forelimb and stretched-out right forelimb, and display an akinetic left forelimb [17]. The EBST is described in detail elsewhere [17], and has been utilized to characterize the biased swing activity of ischemic animals starting at 1 month post-ischemia and extending up to 3 months post-surgery [17]. The animal was lifted 20 times by its tail and the direction of the swing was recorded. An ipsiversive swing activity of = 75% has been suggested as reflective of successful unilateral ischemia or brain insult [17]. The spontaneous rotational test [32] was performed immediately after the animal's recovery from anesthesia following insertion of the embolus. The animal was placed in a chamber made of transparent Plexiglas (40 × 40 × 35.5 cm^3^) and the direction of the animal's rotation was noted over two 5-minute sessions. Two full turns (tight ipsiversive rotations) per minute was considered indicative of a brain insult [17,32]. For the postural bias test and forelimb akinesia test, a semiquantitative scale was used as described elsewhere [33]. The postural tail-hang test involved holding the animal by the tail (similar to the EBST) and noting the positions of the forelimbs. A scale is used for grading the ischemic injury-induced dysfunctions as follows: 0, rats extend straight both forelimbs. No observable deficit; 1, rats keep the left forelimb to the breast and extend straight the right forelimb; 2, rats show decreased resistance to lateral push in addition to behavior in score 1 without circling; 3, rats twist the upper half of their body in addition to behavior in score 2. Finally, the forelimb akinesia test involved pulling each forelimb at 90° away from the body median axis. The return of the forelimb towards the midline was scored as 0 for slow movement with rigidity, 1 for slow movement, and 2 for smooth rapid movement. A score of 1 in the scale for postural bias test or forelimb akinesia test was considered as indicative of CNS dysfunction [17]. These tests were used to characterize any ameliorative effects of DADLE on ischemia-induced dysfunctions from day 0 to day 3 after MCAor surgery.

### Cerebral infarction assay

The volume of infarction was analyzed using the TTC, a histological assay for determining dehydrogenase activity. Animals were euthanized at either 24 h or 72 h after MCAor surgery. Under deep anesthesia (chloral hydrate, 500 mg/kg, i.p.) animals were perfused intracardially with saline. Details of the TTC procedure were described elsewhere [6]. The volume of infarction was measured in each slice and summed using computerized planimetry (PC-based Image Tools software). The volume of infarction was computed as: 2 mm (thickness of the slice) × [sum of the infarction area in all brain slices (mm^2^)] [6]. To minimize artifacts produced by post-ischemic edema in the infarcted area, the infarction area in the ipsilateral hemisphere was indirectly measured by subtracting the non-infarcted area in the ipsilateral hemisphere from the total intact area of the contralateral hemisphere. An additional cohort of ischemic animals (*n *= 4) underwent the same treatment paradigm and were euthanized at 72 h post-stroke for propidium iodide (P-3566, Molecular Probes, Eugene, OR Eugene, OR, USA)Eugene, OR, 20 mg/kg via tail vein) staining to further characterize necrotic cell death.

### p53 mRNA expression

To examine whether DADLE altered programmed cell death or apoptosis, which has been postulated to mediate ischemia-induced cell injury [7,8], we assayed for mRNA expression of the apoptotic marker p53. At 24 h and 72 h after MCAor surgery, randomly selected animals that received either DADLE alone or saline alone were euthanized by rapid decapitation. The striatum was dissected and quickly frozen using dry ice then stored in a -80°C freezer until tissue processing was conducted. Total RNA extraction, Northern blot analysis and hybridization followed those methods described elsewhere [34,35]. Analyses of resulting bands were quantified using a Macintosh computer-based image analysis system (Image, NIH). Densitometrically determined intensities of p-53 mRNA were normalized to 18S rRNA.

In order to further characterize the effects of DADLE on apoptotic cell death, 20 animals were subjected to the same procedures as above, but we focused on only comparing DADLE (*n *= 10) against saline vehicle alone (*n *= 10). Rats were euthanized at 72 h after MCAor surgery. The rats were perfused transcardially with 200 ml of cold phosphate-buffered saline (PBS) and 200 ml of 4% paraformaldehyde in PBS. Brains were removed and post-fixed in the same fixative overnight at 4°C with the subsequent replacement with 30% sucrose in PBS for 72 h. The brains were coronally sectioned at the thickness of 8 μm. Sections were washed 3 times for 5 min in PBS. Sections were then incubated overnight at 4°C with caspase-3 (1:500, Abcam, Cambridge, MA, USACambridge, MA Cambridge, MA Cambridge, MA) or Fas (1:100, Santa Cruz Biotechnology, Santa Cruz, CA, USA) primary antibody and washed 3 times in PBS. Afterwards, sections were incubated with corresponding Cy3-conjugated secondary antibodies (1:1000, Jackson ImmunoResearch Lab, West Grove, PA, USA) for 90 min. Finally sections were washed 3 times for 5 min each in distilled water, and cover-slipped with Gelmount (Biomedia Corp., Foster City, CA, USA). Control studies included exclusion of primary antibody substituted with 10% normal horse serum in PBS. No immunoreactivity was observed in these controls. For morphological analyses, immunoreactive cells in the striatum within the ipsilateral to the stroke hemisphere were examined using a Zeiss LSM510 confocal microscope (Oberkochen, Germany). Specifically, 6 coronal sections at every 300 μm that approximately captured the ischemic striatum (AP -2.0 to +2.0 mm from the bregma) were examined from each rat and the number of positive cells was counted in each 6 high power fields and the averages were used for the statistical analyses. Alternate sections from the additional cohort of animals used for propidium iodide (see *Cerebral infarction assay *above) were processed for caspase-3 immunofluorescent imaging to further reveal apoptotic cell death.

### Enzyme-linked immunosorbent assay

To examine possible non-opioid effects of DADLE, we conducted assays of expression of neurotrophic factors (GDNF and NGF) that have been previously shown to be protective against ischemia [6,36]. Enzyme-linked immunosorbent assay (ELISA) was conducted on ischemic animals injected either with DADLE or with saline (*n *= 6 per group). Following the same drug dosing regimen mentioned above, animals were rapidly decapitated, their brains removed and striatal and frontal cortical tissues quickly dissected. Brain homogenization and supernatant acidification followed those methods described elsewhere [37] with minor modifications. Protein concentrations were measured by using the BCA Kit (Pierse, Rockford, IL, USA). For the measurement of GDNF, mouse monoclonal anti-GDNF antibody (R & D Systems, Inc., Minneapolis, MN, USA) was used as a capture antibody and biotinylated goat anti-GDNF antibody (R & D Systems, Inc., Minneapolis, MN, USA) was used as a detection antibody. For the measurement of NGF, the NGF Emax TM ImmunoAssay system (Promega Cooperation, Madison, WI, USA) was used. The THERMOmax 96 well microplate reader (Molecular Devices Corp., Sunnyvale, CA, USA) was used to measure the optical densities.

### Statistical analyses

ANOVA followed by *post hoc *test using Fisher's protected least significant difference (PLSD) was used to reveal statistical significance in behavioral, mRNA, ELISA, and histological data. A statistically significant difference was pre-set at *P <*0.05.

## Abbreviations

ANOVA: analysis of variance; CNS: central nervous system; DADLE: [D-ala2,D-leU5]enkephalin; EBST: elevated body swing test; ELISA: enzyme-linked immunosorbent assay; GDNF: glial cell line-derived neurotrophic factor; MCA: middle cerebral artery; MCAor: occlusion and reperfusion surgery of the middle cerebral artery; NGF: nerve growth factor; PBS: phosphate-buffered saline; PLSD: protected least significant difference; TTC: triphenyltetrazolium.

## Authors' contributions

CVB, TH, PRO, TPS and YW conceived and designed the experiments, analyzed and interpreted the data, provided intellectual input, read and approved the final version of the manuscript,
